# Alkaloids from Stems of *Esenbeckia leiocarpa* Engl. (Rutaceae) as Potential Treatment for Alzheimer Disease 

**DOI:** 10.3390/molecules15129205

**Published:** 2010-12-13

**Authors:** Elaine Monteiro Cardoso-Lopes, James Andreas Maier, Marcelo Rogério da Silva, Luis Octávio Regasini, Simone Yasue Simote, Norberto Peporine Lopes, José Rubens Pirani, Vanderlan da Silva Bolzani, Maria Cláudia Marx Young

**Affiliations:** 1 Section of Plant Physiology and Biochemistry, Institute of Botany, Box 3005, 01061-970, São Paulo, SP, Brazil; 2 Institute of Chemistry, São Paulo State University, Box 355, 14801-970, Araraquara, SP, Brazil; 3 Department of Chemistry and Physics, São Paulo University, 14040-903, Ribeirão Preto, SP, Brazil; 4 Department of Botany, Biosciences Institute, São Paulo University, Box 11.461, 05422-970, São, Paulo, SP, Brazil

**Keywords:** *Esenbeckia leiocarpa*, Rutaceae, alkaloids, acetylcholinesterase inhibitors

## Abstract

*Esenbeckia leiocarpa* Engl. (Rutaceae), popularly known as *guarantã, goiabeira*, is a native tree from Brazil. Bioactivity-guided fractionation of the ethanol stems extract afforded the isolation of six alkaloids: leiokinine A, leptomerine, kokusaginine, skimmianine, maculine and flindersiamine. All isolated compounds were tested for acetyl cholinesterase inhibition, *in vitro* and displayed anticholinesterasic activity. The alkaloid leptomerine showed the highest activity (IC_50_ = 2.5 μM), similar to that of the reference compound galanthamine (IC_50_ = 1.7 μM). The results showed for the first time the presence of alkaloids leptomerine and skimmianine in *E. leiocarpa* (Engl.) with potent anticholinesterasic activity.

## 1. Introduction

The genus *Esenbeckia* comprises 30 species distributed in Tropical America [1]. Chemical studies of *Esenbeckia* spp. showed that they synthesize a variety of secondary metabolites, particularly quinolinic, quinolonic and indolic alkaloids and furocoumarins, and these compounds were considered chemical markers of this genus [2,3,4].

*Esenbeckia leiocarpa* Engl. (Rutaceae), native from Brazil, is an ornamental tree widely distributed in Brazilian Dense Ombrophilous Atlantic Forest from South of Bahia to São Paulo states and is commonly known as *guarantã* (SP), *goiabeira* (BA), *antã-forte* and *guarataia* (ES) [5]. 

Previous studies on *E. leiocarpa* have been reported in the literature, and various compounds have been isolated from it, such as the alkaloids kokusaginine, flindersiamine, maculine, dictamnine, 4-methoxy-2-(3´-pentyl)quinoline, 1,4-dihydro-1-methyl-2-(3´-pentyl)quinolin-4-one, leiokinine A and leiokinine B, twelve indole derivatives, three lignans, one coumarin, two amides and the methyl 4-isoprenyloxy-*trans*-cinnamate [3,6,7].

Alzheimer´s disease (AD) is the most common cause of progressive cognitive dysfunction that results from a deficiency in cholinergic activity in brain [8]. Several plant-derived drugs (rivastigmine and galanthamine) that inhibit acetylcholinesterase (AChE) can be used to treat early stages of AD, since these compounds increase the endogenous levels of acetylcholine to boost cholinergic neurotransmission. In a recent review, 260 chemically defined natural molecules were evaluated for acetylcholinesterase inhibition. The compounds tested were classified in alkaloids (139), monoterpenes (27), coumarins (18), triterpenes (17), flavonoids (14), benzenoids (13), diterpenes (8), oxygen heterocycles (5), sesquiterpenes (5), stilbenes (3), lignans (2), sulfur compounds (2), proteids (2), polycyclic (1), quinoid (1), benzoxazinone (1), carotenoid (1) and alycyclic (1) [9,10,11].

Strong efforts to discover new acetylcholinesterase inhibitors from a vast number of plant species were realized in our laboratory. In a screening of 58 extracts from native Brazilian plants, the ethanolic crude extract of the stems of *Esenbeckia leiocarpa* showed a strong acetylcholinesterase inhibition and was chosen for bioassay-guided fractionation in order to isolate the biologically active compounds and evaluate the acetylcholinesterase inhibition of these compounds.

## 2. Results and Discussion

### 2.1. Effect of crude extract and hexane and alkaloid fractions on acetylcholinesterase inhibition

**Figure 1:**
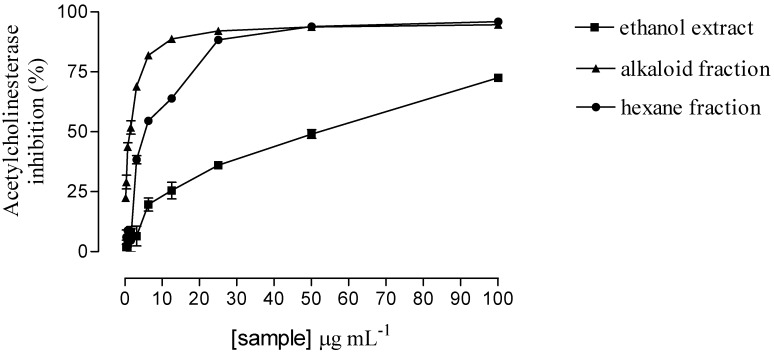
Acetylcholinesterase inhibition by ethanol extract, alkaloid and hexane fractions (0.2 to 100.0 μg/mL).

The crude ethanol extract of *Esenbeckia leiocarpa* stems inhibited 91.1 ± 0.2% of acetylcholinesterase activity when tested at 200 μg/mL. Acid-base partition was used to separate the crude extract (35.8 g) of *Esenbeckia leiocarpa* into two fractions, hexane and chloroform (Alkaloid) and the yield of each fraction was 0.64% and 3.30%, respectively. The ethanol extract, hexane and alkaloid fractions of *Esenbeckia leiocarpa* inhibited acetylcholinesterase activity with an IC_50_ 50.7 μg/mL, 6.0 μg/mL and 1.6 μg/mL, respectively (Figure 1). The chloroform fraction, with positive result for alkaloids (Dragendorff´s and iodoplatinate reagents), exhibited highest level of AChE inhibition and was selected for further purification. 

### 2.2. Chromatographic profile of alkaloid fraction

**Figure 2:**
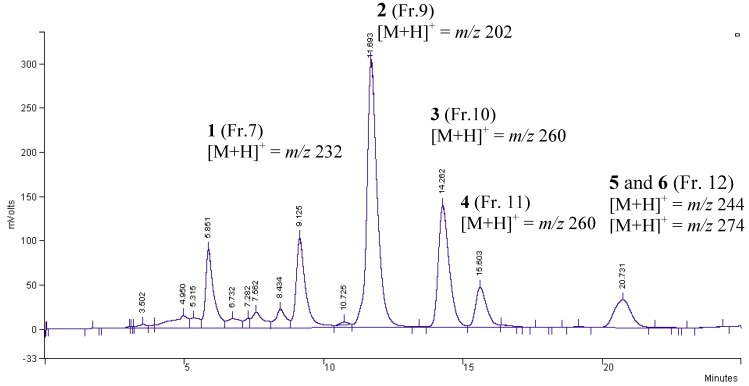
Analytical chromatographic profile of alkaloid fraction from *E. leiocarpa* by HPLC and their respectives molecular ion peaks [M+H]^+^ data in the ESI-MS.

The analytical chromatographic (HPLC) analyses of alkaloid fraction (105.0 mg) from *E. leiocarpa* afforded 14 fractions from which six major compounds were identified. They were fractionated by chromatographic procedure and the anticholinesterasic activity of each fraction was tested by TLC bioassay as described by Marston *et al*. [12]. The fractions 7, 9, 10, 11 and 12 presented strong anticholinesterasic activity on TLC bioassay (results not showed) and retention times (*t*_R_) of 9.12 min, 11.69 min, 14.26 min, 15.60 min and 20.73 min, respectively (Figure 2).

### 2.3. Isolation and identification of alkaloids from stems of E. leiocarpa

**Figure 3:**
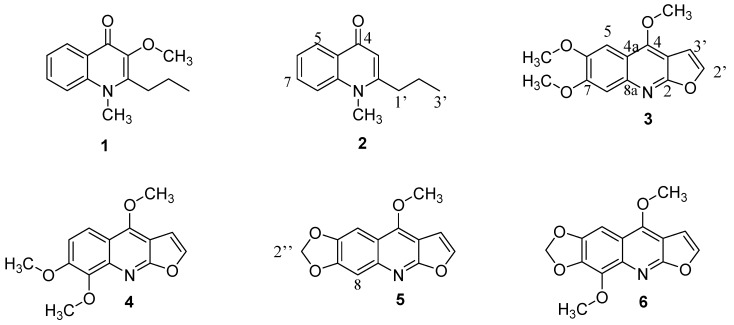
Chemical structures of alkaloids with anticholinesterasic activities, ALK 1 to 6. The alkaloids isolated are: **1** – leiokinine A, **2** – leptomerine, **3** – kokusaginine, **4** – skimmianine, **5** – maculine, **6** – flindersiamine.

The alkaloid fraction of *E. leiocarpa* (m = 105.0 mg) was dissolved into MeOH (10 mg/mL) and submitted to RP-HPLC using acetonitrile-water-methanol (10:45:45) as mobile phase, with UV detection at 242 nm and flow rate of 8 mL/min, affording 14 fractions (Fractions 1–14). From preparative HPLC, fractions Fr. 7 (*t*_R_ = 15.4 min) and Fr. 9 (*t*_R_ = 19.3 min) were identified as two 4-quinolinone alkaloids, leiokinine A (**1**, 5.8 mg) [3] and leptomerine (**2**, 16.0 mg) [13], respectively. Fractions Fr. 10 (*t*_R_ = 24.5 min) and Fr. 11 (*t*_R_ = 26.2 min) yielded two furoquinoline alkaloids, kokusaginine (**3**, 5.7 mg) [14] and skimmianine (**4**, 12.3 mg) [15], respectively. In addition, fraction Fr. 12 (*t*_R_ = 34.3 min) afforded a mixture of two others furoquinoline alkaloids (3.2 mg), maculine (**5**) [16] and flindersiamine (**6**) [6] (Figure 3). Their identification was based on analysis of 1D and 2D NMR experiments, as well as by comparison with literature data, ^13^C-NMR data of compounds **1**, **2**, **3** and **4** and ^1^H-NMR data of compounds **5** and **6** (see Experimental section).

### 2.4. Anticholinesterasic activity of alkaloids

The alkaloids maculine, flindersiamine, kokusaginine, leptomerine, leiokinine A and skimmianine identified in this work were previously isolated from other *Esenbeckia* species [1,17,18,19]. The alkaloids leptomerine and skimmianine have already been isolated from *Haplophyllum heptomerum* (Rutaceae) in 1986 [13]. However, this is the first occurrence of leptomerine and skimmianine in *Esenbeckia leiocarpa* (Engl.). 

**Figure 4:**
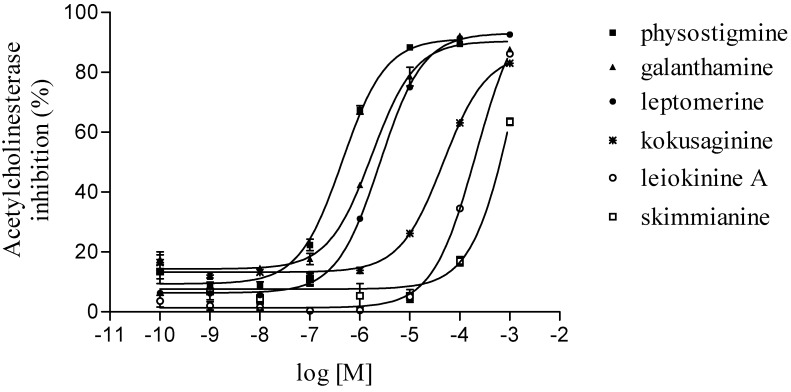
Acetylcholinesterase inhibition of physostigmine, galanthamine, leptomerine, kokusaginine, leiokinine A and skimmianine (10^-10^ to 10^-3^ M).

To date, no study on evaluation of acetylcholinesterase activity was performed with species of the genus *Esenbeckia*. In the present paper, the alkaloids isolated from stems of *E. leiocarpa*, leiokinine A and skimmianine presented acetylcholinesterase inhibition with IC_50_ values of 0.21 mM and 1.4 mM, respectively. While leptomerine and kokusaginine showed IC_50_ values of 2.5 μM and 46 μM, respectively (Figure 4). The alkaloids leptomerine and kokusaginine had a potent effect anticholinesterasic similar to reference compounds galanthamine and physostigmine that have 1.7 μM and 0.4 μM values of IC_50_, respectively.

Previous biological studies with chloroform extract from leaves of *E. leiocarpa* demonstrated weak antifeedant activity against the pink bollworm, *Pectinophora gossypiella,* this activity was attributed to alkaloids leiokinine A and leiokinine B [3]. On the other hand, the alkaloids isolated from leaves of *E. belizencis* Lundell were tested in the brine shrimp toxicity assay and revealed that kokusaginine have mildly toxicity (LC_50 _= 367 ppm) and flindersiamine did not showed toxicity (LC_50 _> 1,000 ppm) [20]. The alkaloids maculine, kokusaginine, skimmianine and flindersiamine demonstrated weak mutagenic activity [21,22]. In another species of Rutaceae, *Raulinoa echinata* Cowan, these same alkaloids showed antifungal activity against *Leucoagaricus gongylophorus*, a symbiotic fungus of leaf-cutting ants [23]. 

## 3. Experimental

### 3.1. Plant material

The stems of *Esenbeckia leiocarpa* Engl. were collected in January 2007 from a specimen cultivated at the “Cidade Universitária - Armando Salles de Oliveira- CUASO”, USP, São Paulo State, Brazil, and identified by Dr. José Rubens Pirani. A voucher specimen (SPF 1169) was deposited at the Herbarium of the Departamento de Botânica, Instituto de Biociências, USP, São Paulo, SP, Brazil.

### 3.2. Extraction and isolation

The dry stems of *Esenbeckia leiocarpa* (1000 g) were powdered and extracted with ethanol (5 × 1.5 L) using a maceration method. The ethanol extract (35.8 g) was re-dissolved in aqueous solution (HCl 0.1 M, 2 × 120 mL) and the insoluble portion was filtered off. The acid solution was partitioned with hexane (7 × 60 mL) yielding a hexane fraction (0.230 g). The acid aqueous fraction was basified with NH_4_OH (pH 10) and partitioned with CHCl_3_ (7 × 60 mL) yielding an active alkaloid fraction (1.182 g). All the fractions were tested in biological test for acetylcholinesterase inhibitor activity. 

### 3.3. General methods for compounds identification

The alkaloid fraction was analyzed in HPLC Varian Pro Star 310 system equipped with a 20 μL injection loop. The column used was a Phenomenex C-18 (250 × 4.6 mm) maintained at 20 °C. Twenty μL of the sample (1 mg/mL) was injected, the mobile phase (1 mL/min) was ACN: MeOH: H_2_O (10:45:45). Preparative HPLC was carried out using Varian Prep-Star 400 system using a Phenomenex C-18 (250 mm × 20.0 mm) preparative column. HPLC analysis of alkaloid fraction afforded 14 fractions. Fractions 7, 9, 10, 11 and 12 were identified by NMR analyses. The 1D – (^1^H, ^13^C and DEPT) and 2D (^1^H-^1^H *g*COSY, *g*HMQC and *g*HMBC) NMR experiments were recorded on a Varian INOVA 500 spectrometer (11.7 T) at 500 MHz (^1^H) and 125 MHz (^13^C), using the CDCl_3_ as an internal standard.

*Leiokinine A* (**1**). ^1^H-NMR [CDCl_3_, δ (ppm), *J* (Hz)]: δ 2.97 (H-C1`), 1.68 (H-C2`), 1.10 (H-C3`), 8.53 (dd, *J* = 8.0 and 1.5 Hz, H-C5), 7.36 (tl, *J* = 8.0 Hz, H-C6), 7.52 (dl, *J* = 8.0 Hz, H-C7), 7.65 (dt, *J* = 8.0 and 1.5 Hz, H-C8), 3.82 (s, H-N-CH_3_), 3.94 (s, H-C3-OCH_3_) [3]. ^13^C-NMR [CDCl_3_]: δ 29.48 (C-1`), 22.23 (C-2`), 14.15 (C-3`), 148.45 (C-2), 140.01 (C-3), 172.01 (C-4), 141.07 (C-5), 126.52 (C-6), 123.11 (C-7), 131.70 (C-8), 115.29 (C-9), 126.77 (C-10), 34.93 (N-CH_3_), 60.22 (3-OCH_3_) [3].

*Leptomerine* (**2**). ^1^H-NMR [CDCl_3_, δ (ppm), *J* (Hz)]: δ 1.60 (m, H-C1’), 1.60 (m, H-C2’), 0.85 (t, H-C3’), 6.36 (s, H-C3), 8.41 (dd, H-C5), 7.34 (t, H-C6), 7.52 (d, H-C7), 7.63 (d, H-C8), 3.77 (s, H-N-CH_3_). ^13^C-NMR [CDCl_3_]: δ 27.6 (C-1’), 27.6 (C-2’), 11.6 (C-3’), 109.0 (C-3), 177.23 (C-4), 142.14 (C-5), 126.57 (C-6), 123.70 (C-7), 132.18 (C-8), 115.9 (C-9), 126.7 (C-10), 34.4 (N-CH_3_) [13].

*Kokusaginine* (**3**). ^1^H-NMR [CDCl_3_, δ (ppm), *J* (Hz)]: δ 7.53 (d, *J* = 2.5 Hz, H-C2’), 7.02 (d, *J* = 2.5 Hz, H-C3’), 7,47 (s, H-C5), 7,40 (s, H-C8), 4,41 (s, H-C4-OCH_3_), 4,03 (s, H-C6-OCH_3_), 4.02 (s, H-C7-O CH_3_) [21]. ^13^C-NMR [CDCl_3_]: δ 142.7 (C-2’), 104.8 (C-3’), 112.9 (C-3), 102.4 (C-4a), 100.2 (C-5), 105.8 (C-8), 142.7 (C-8a), 59.1 (4-OCH_3_), 56.2 (6-OCH_3_), 56.0 (7-OCH_3_) [21].

*Skimmianine* (**4**). ^1^H-NMR [CDCl_3_, δ (ppm), *J* (Hz)]: δ 7,54 (d, *J* = 2.5 Hz, H-C2’), 7.00 (d, *J* = 2.5 Hz, H-C3’), 7.95 (d, *J* = 9.5 Hz, H-C5), 7.17 (d, *J* = 9.5 Hz, H-C6), 4.39 (s, H-C4-OCH_3_), 4.06 (s, H-C7-OCH_3_), 3.97 (s, H-C8-OCH_3_) [14,21]. ^13^C-NMR [CDCl_3_]: δ 143.2 (C-2’), 104.7 (C-3’), 118.2 (C-5), 112.4 (C-6), 59.8 (4-OCH_3_), 56.8 (7-OCH_3_), 61.7 (8-OCH_3_) [15,21].

*Maculine* (**5**). ^1^H-NMR [CDCl_3_, δ (ppm), *J* (Hz)]: δ 7.56 (d, *J* = 2.4 Hz, H-C2’), 7.03 (d, *J* = 2.4 Hz, H-C3’), 7.46 (s, H-C5), 7.21 (s, H-C8), 6.07 (s, H-C2”), 4.41 (s, H-C4-OCH_3_) [16,21].

*Flindersiamine* (**6**). ^1^H-NMR [CDCl_3_, δ (ppm), *J* (Hz)]: δ 7.52 (d, *J* = 2.4 Hz, H-C2’), 6.99 (d, *J* = 2.4 Hz, H-C3’), 7.19 (s, H-C5), 6.00 (s, H-C2”), 4.39 (s, H-C4-OCH_3_), 4.19 (s, H-C8-OCH_3_) [21].

### 3.4. Anticholinesterasic assay

Acetylcholinesterase activity was measured using a 96-well microplate reader [24] based on Ellman´s method [25]. In the 96-well plates, 25 μL of 15 mM acetylthiocholine iodide (ATCI) in water, 125 μL of 3 mM DTNB in buffer C, 50 μL of buffer B, 25 μL of plant extract, alkaloid or hexane fractions (0.2 to 100.0 μg mL^-1^) were added and the absorbance was measured at 405 nm every 30 s for three times. Then 25 μL of 0.22 U/mL of the enzyme were added and the absorbance was again read every 10 min for two times. The percentage of inhibition was calculated by comparison with the rates for the sample to a blank (10% MeOH in Buffer A). The following buffers were used. Buffer A: 50 mM Tris-HCl, pH 8; buffer B: 50 mM Tris-HCl, pH 8, containing 0.1% bovine serum albumin V fraction (BSA); buffer C: 50 mM Tris-HCl, pH 8, containing 0.1 M NaCl and 0.02 M MgCl_2_·6H_2_O.

### 3.5. Statistical analysis of data

Data were presented as means ± SEM of experiments realized at least in triplicate. The IC_50_ values were calculated by means of regression analysis.

## 4. Conclusions

The alkaloids kokusaginine, flindersiamine, maculine and leiokinine A were previously isolated from *E. leiocarpa*. On the other hand, this is the first occurrence of leptomerine and skimmianine in *Esenbeckia leiocarpa*. There are many compounds identified from higher plants with acetylcholinesterase inhibition activity as huperzine A, galanthamine isolated from *Huperzia serrata* (Thunb.) Trev. [26] and *Galanthus nivalis* Lumikello, respectively, and used for dementia treatment.

Some biological activities have been reported for the alkaloids isolated in this work, but none have been found to be related to acetycholinesterase inhibition. This work demonstrated that these alkaloids presented potent anticholinesterasic activity and must be tested in pharmacological models *in vivo* to determine if they have activity on memory of mice and pre clinical studies before human application. 
